# Nematicidal Activity of Essential Oils on a Psychrophilic *Panagrolaimus* sp. (Nematoda: Panagrolaimidae)

**DOI:** 10.3390/plants9111588

**Published:** 2020-11-17

**Authors:** Violeta Oro, Slobodan Krnjajic, Marijenka Tabakovic, Jelena S. Stanojevic, Snezana Ilic-Stojanovic

**Affiliations:** 1 Department of Plant Diseases, Institute for Plant Protection and Environment, 11000 Belgrade, Serbia; 2 Institute for Multidisciplinary Research, University of Belgrade, 11000 Belgrade, Serbia; slobodan.krnjajic@imsi.bg.ac.rs; 3 Agroecology and Cropping Practices Group, Maize Research Institute, 11000 Belgrade, Serbia; mtabakovic@mrizp.rs; 4 Faculty of Technology Leskovac, University of Nis, 16000 Leskovac, Serbia; jstanojevic@tf.ni.ac.rs (J.S.S.); snezanai@tf.ni.ac.rs (S.I.-S.)

**Keywords:** essential oils, *Panagrolaimus* sp., LC50, aldehydes

## Abstract

Essential oils (EOs) have historically been used for centuries in folk medicine, and nowadays they seem to be a promising control strategy against wide spectra of pathogens, diseases, and parasites. Studies on free-living nematodes are scarce. The free-living microbivorous nematode *Panagrolaimus* sp. was chosen as the test organism. The nematode possesses extraordinary biological properties, such as resistance to extremely low temperatures and long-term survival under minimal metabolic activity. Fifty EOs from 22 plant families of gymnosperms and angiosperms were tested on *Panagrolaimus* sp. The aims of this study were to investigate the in vitro impact of EOs on the psychrophilic nematode *Panagrolaimus* sp. in a direct contact bioassay, to list the activity of EOs based on median lethal concentration (LC50), to determine the composition of the EOs with the best nematicidal activity, and to compare the activity of EOs on *Panagrolaimus* sp. versus plant parasitic nematodes. The results based on the LC50 values, calculated using Probit analysis, categorized the EOs into three categories: low, moderate and highly active. The members of the laurel family, i.e., *Cinnamomum cassia* and *C. burmannii*, exhibited the best nematicidal activity. Aldehydes were generally the major chemical components of the most active EOs and were the chemicals potentially responsible for the nematicidal activity.

## 1. Introduction

Nematodes are mostly microscopic size invertebrates that inhabit terrestrial and aquatic areas. Beside their significant economic importance as human, animal and plant parasites, they can also be beneficial, free-living microbivorous organisms. It has been estimated that about 2.5 million tons of pesticides are used on crops each year [1]. Such a practice has resulted in the decline of many beneficial organisms, such as nitrogen-fixing soil bacteria [2], blue-green algae [3], mycorrhizal fungi [4], water fishes [5], aquatic mammals [6], and birds [7]. In addition, the pesticide residues in food and water are massive long-term threats for human health at a global level. According to the European legislation (Regulation (EC) no. 1107/2009), the application of non-chemical and natural alternatives should be the first choice in plant protection and integrated pest management. The Regulation requires that substances or products produced or placed on the market do not have any harmful effect on human or animal health or any unacceptable effects on the environment, such as an impact on non-target species and impact on biodiversity and the ecosystem. 

The use of essential oils (EOs) is known from folk medicine centuries ago [8]. Nowadays, it seems to be a promising control strategy against different nematode plant and animal parasites (*Bursaphelenchus xylophilus*, *Cooperia* spp., *Ditylenchus dipsaci*, *Haemonchus contortus*, *Meloidogyne chitwoodi*, *M. incognita*, *M. javanica*, *Oesophagostomum* spp., *Pratylenchus penetrans*, *Steinernema feltiae*, *Trichostrongylus* spp., *Tylenchulus semipenetrans*) [9,10,11,12,13,14,15,16,17,18,19,20,21,22,23]. Microbivorous nematodes contribute to decomposition of organic matter and the release of nutrients for plant uptake [24], which makes them important components of the soil microfauna. A free-living, microbivorous nematode, *Panagrolaimus* sp. Fuchs, was chosen as a test organism. *Panagrolaimus* sp. is a non-target organism, easy to maintain, does not have a complex life cycle, in contrast to plant parasitic nematodes [25], and possesses some extraordinary biological properties. This nematode, known as the Antarctic nematode, is famous for its resistance to intracellular freezing and extremely cold environmental conditions [26]. *Panagrolaimus* aff. *detritophagus* is the first viable multicellular organism, isolated from 30,000–40,000-year-old permafrost deposits [27].

This study aims to: (i) investigate the in vitro impact of EOs on the psychrophilic nematode *Panagrolaimus* sp. in a direct contact bioassay, (ii) list the activity of the EOs based on median lethal concentration (LC50), (iii) determine the composition of the EOs with the best nematicidal activity, and (iv) compare the activity of EOs on the non-target panagrolaimid nematode versus plant parasitic nematodes.

## 2. Results

The nematicidal activity of the EOs on the *Panagrolaimus* sp. juveniles are presented in Table 1.

The results based on the median lethal concentration (LC50) of 50 EOs are in range from 0.033 to 5.641 µL/mL. The list of all EOs could be divided into three groups. The first group is made up of those with LC50 values higher than 1 µL/mL, the next group with LC50 values in the range 0.1 to 1 µL/mL, and the last group with the LC50 values lower than 0.1 µL/mL. The lowest nematicidal impact is observed in the first group containing EOs from different plants with a significant content of gymnosperms, represented by the families Pinaceae and Cupressaceae. In the same group are some members of angiosperms, such as Burseraceae, Myrtaceae, etc. The second group with a moderate nematicidal effect on the panagrolaimid nematode had EOs originating mainly from the family Lamiaceae and some representatives from individual families. This study demonstrates, for the first time, the nematicidal activity of *Turnera diffusa*, *Taxandria fragrans* and *Uncaria tomentosa* EOs originating from the families *Passifloraceae*, *Myrtaceae*, and *Rubiaceae*, respectively. The best nematicidal activity among the three species was exhibited by *Uncaria tomentosa* EO, with an LC50 of 0.222 µL/mL.

The highest nematicidal impact was observed with three representatives from the family Lauraceae, namely *Litsea citrata*, *Cinnamomum cassia*, and *C. burmannii*, two representatives from the family Apiaceae—i.e., *Foeniculum vulgare* and *Coriandrum sativum*—and the single species *Cymbopogon flexuosus* from the family Poaceae, with LC50 values ranging from 0.033 to 0.091 µL/mL. The best nematicidal effect on panagrolaimid nematodes was shown by *Cinnamomum. burmannii* EO, extracted from the bark. The chemical composition of the EOs with the best nematicidal performance on *Panagrolaimus* sp., with the retention time (RT, in minutes) and the retention indices obtained experimentally and from the literature (RI^exp^ and RI^lit^, respectively), are given in Table 2, Table 3, Table 4, Table 5, Table 6 and Table 7. 

According to the gas chromatography/mass spectrometry (GC/MS) result obtained, 24 compounds were identified, representing 99.2% of total *Litsea. citrata* EO composition. The main components belong to oxygen-containing monoterpenes (contributing 84.3%), with citral—i.e., geranial (43.4%) and neral (32.2%)—as their representatives present in the highest percentage. They are followed by monoterpene hydrocarbons with 12.2% and limonene as their representative with a contribution of 9.4%, and sesquiterpene hydrocarbons ((E)-caryophyllene, β-elemene and α-humulene) with a contribution of 1.9% to the total EO composition (Table 2).

According to the results of GC/MS analysis of *Foeniculum. vulgare* EO, 18 compounds were identified, representing 99.1% of total EO composition. The main components were aromatic compounds (78.5%), with (E)-anethole as their representative present in the highest percentage (74.3%), followed by oxygen-containing monoterpenes (14.8%) and their representatives fenchone (2.1%) and carvone (2.1%), and monoterpene hydrocarbons (5.8%) with the highest amount of limonene (2.3%) (Table 3). 

The results of the GC/MS analysis of the *Cymbopogon. flexuosus* EO revealed 32 compounds, representing 97.3% of total EO composition. The main components were oxygen-containing monoterpenes (86.3%) with geranial and neral (citral), contributing 40.3% and 30.9%, respectively, geranyl acetate and geraniol (5.4% and 4.5%, respectively), followed by sesquiterpene hydrocarbons (4.0%) and their representative (E)-caryophyllene (2.1%) (Table 4).

GC/MS analysis of the *Coriandrum. sativum* EO resulted in identifying 29 compounds, representing 97.5% of total EO composition. The main components were aldehydes (contributing 51.8%), with (2E)-decenal as their representative, followed by aliphatic alcohols (among which (2E)-decen-1-ol was present in the highest percentage of 16.3%) and oxygen-containing monoterpenes (21.7%) with linalool as the most abundant compound (18.4%) (Table 5). 

The GC/MS analysis of *Cinnamomum. cassia* EO revealed 32 compounds, representing 99.3% of total EO composition. The main components belong to the group of aromatic compounds, contributing 91.8%, followed by sesquiterpene hydrocarbons (6.7%). (E)-Cinnamaldehyde and eugenol acetate were identified as the representatives of aromatic compounds contributing 76.7% and 7.4%, respectively. On the other hand, δ-cadinene with a contribution of 6.2% to the total EO composition, was identified as a representative compound from the sesquiterpene hydrocarbons group (6.7%) (Table 6).

According to the GC/MS results, 43 compounds, representing 98.1% of total *C. burmanii* EO composition, were identified. The main components belong to aromatic compounds (contributing 84.5% in the total EO composition), with (E)-cinnamaldehyde as their representative (80.5%). They are followed by sesquiterpene hydrocarbons (7.0%) with δ-cadinene and α-copaene as their members present in the amounts of 1.7% and 1.5%, respectively and oxygenated monoterpenes (5.5%) with α-terpineol (1.9%) as their main representative (Table 7).

## 3. Discussion

Essential oils with the highest nematicidal activity, demonstrated in this study, have been reported to be efficient against wide spectra of pathogens, diseases, and parasites.

The *Litsea citrata* EO showed antibacterial, antifungal, acaricidal, and nematicidal activities. The fruit essential oil of *Litsea cubeba* (*syn. Litsea citrata*) exhibited antibacterial activity against *Bacillus cereus*, *Staphylococcus aureus*, *Vibrio parahaemolyticus*, and *Klebsiella pneumoniae* [28]. As an antifungal agent it was effective against *Candida krusei* and *C. guilliermondii* but did not act against *C. albicans*, *C. tropicalis* and *C. parapsilosis* [29]. The *Litsea cubeba* EO had acaricidal activity against house dust mites, *Dermatophagoides farinae* and *D. pteronyssinus*, and stored food mites, *Tyrophagus putrescentiae* [30]. The LC50 values of ajowan, allspice and litsea were 0.431, 0.609 and 0.504 mg/mL, respectively, and exhibited good nematicidal activity against *B. xylophilus* [31]. Citral, i.e., geranial and neral, were the main compounds in the *Litsea citrata* EO in this study. Citral (3,7-dimethyl-2,6-octadienal) is the monoterpene aldehyde representing natural mixture of the two geometric isomers: geranial (trans-isomer) with a strong lemon odor and neral (cis-isomer) with a lemon odor that is less intense and sweeter than geranial [32].

The *Foeniculum vulgare* EO exhibited antifungal, antibacterial, antiviral, and nematicidal activities. In the inverted petriplate method, the volatile oil showed complete zone inhibition against *Aspergillus niger*, *A. flavus*, *Fusarium graminearum*, and *F. moniliforme* at a 6-µL dose [33]. Hot water extracts of fennel seeds was effective against *Enterococcus faecalis*, *S. aureus*, *Escherichia coli*, *Pseudomonas aeruginosa*, *Salmonella typhimurium*, *S. typhi*, and *Shigella flexneri* [34]. The DNA virus *Herpes simplex* type-1 (HSV-1) and the RNA virus parainfluenza type-3 (PI-3) were inhibited by the *Foeniculum. vulgare* EO [35]. Essential oils of *Carum carvi*, *F. vulgare*, *Mentha rotundifolia*, and *M. spicata* showed the highest nematicidal activity against *M. javanica* juveniles [9].

Trans-anethole was the most abundant compound in the *Foeniculum vulgare* EO, reaching 74% of the total identified constituents. Propenylbenzenes, such as anethole, were reported to be mutagenic for *Salmonella* tester strains and also carcinogenic in the induction of hepatomas in B6C3F1 mice and skin papillomas in CD-1 mice [36].

The *Cymbopogon flexuosus* lemongrass EO was reported to have antibacterial, antifungal and anti-inflammatory activity. The EO from *C. flexuosus* exhibited an antimicrobial effect against *B. subtilis*, *Staphylococcus aureus*, *A. flavus* and *A. fumigatus* [37]. The lemongrass (*C. flexuosus*) inflorescence EO was inhibitory to *Pyricularia oryzae*, *Dreshlera oryzae*, *A. niger* and *Penicillium italicum* [38]. Lemongrass EO, which has citral as its main component, has exhibited an anti-inflammatory effect in both animal and human cells [39]. In this study, the content of the lemongrass EO’s major compounds, geranial and neral, was similar to their content in the *L. citrata* EO with slightly lower amounts—40.3% and 30.9%, respectively.

The *Coriandrum sativum* EO exhibited antifungal, antibacterial, insecticidal, and nematicidal activities. The *Coriandrum sativum* EO showed excellent antifungal activity against seedborne pathogens *P. oryzae*, *Bipolaris oryzae*, *Alternaria alternata*, *Tricoconis padwickii*, *Drechslera tetramera*, *D. halodes*, *Curvularia lunata*, *F. moniliforme*, and *F. oxysporum* [40]. The methanolic extract of *C. sativum* showed antibacterial activity against *E. coli*, *P. aeruginosa*, *S. aureus*, and *K. pneumoniae* [41]. The leaf oil had significant toxic effects against the larvae of *Aedes aegypti* with an LC50 value of 26.93 ppm and an LC90 value of 37.69 ppm, and the stem oil has toxic effects against the larvae of *A. aegypti* with an LC50 value of 29.39 ppm and an LC90 value of 39.95 ppm [42]. Among the 28 plant EOs tested for their nematicidal activities against the pine wood nematode, *B. xylophilus*, the best nematicidal activity was achieved with the EO of coriander [43]. In this study, the major compound in the *C. sativum* EO was trans-2-decenal with 28.2%, affiliated to the group of medium-chain aldehydes. Aliphatic aldehydes (mainly C_10_–C_16_ aldehydes), with their unpleasant odor, are the main components of the volatile oil from the fresh herb [44]. Aldehydes present in the coriander EO are important biologically active substances due to their possible toxic activity against tropical mosquitoes transmitting dangerous illnesses [45].

The *Cinnamomum cassia* EO exhibited antimicrobial, antiviral, insecticidal, and nematicidal activities. The cassia EO acted as fungal growth inhibitor against *A. flavus* and *A. oryzae* [46] and as a bacterial inhibitor of *S. aureus* and *E. coli* [47]. The silver nanoparticles derived from cinnamon extract enhanced the antiviral activity and were found to be effective against highly pathogenic avian influenza virus subtype H7N3, when incubated with the virus prior to infection and introduced to cells after infection [48]. The chloroform extract from *C. cassia* was the most effective against *Dermestes maculatus* larvae, the pest of Egyptian mummies [8]. Cassia oil was efficient against *Sitophilus zeamais* [49], and the booklice *Liposcelis bostrychophila* [50]. As judged by the 24-h LC50 values, two cassia oils (0.084–0.085 mg/mL) and four cinnamon oils (0.064–0.113 mg/mL) were toxic toward adult *B. xylophilus* [51].

As opposed to cassia, *Cinnamomum burmannii* EO has been less studied. The *Cinnamomum burmannii* EO exhibited significant antibacterial properties against five common foodborne pathogenic bacteria, namely, *B. cereus*, *L. monocytogenes*, *S. aureus*, *E. coli*, and *Salmonella anatum* [52].

The major component of cinnamon bark EO is (E)-cinnamaldehyde. In the contact with bacterial membrane, cinnamaldehyde causes the loss of membrane functionality or the loss of channel proteins in the membrane, resulting in death of bacterial cells [53]. Besides this, (*E*)-cinnamaldehyde was significantly more effective than its corresponding acid (cinnamic acid) and alcohol (cinnamyl alcohol) and could be used as a fumigant with contact action in the control of house dust mites, *D. farinae* and *D. pteronyssinus* [54].

It has been emphasized that the major components play important roles in the toxicity of EOs [31,42,55] and the majority of them belong to the class of terpenes. Terpenes are the largest class of secondary metabolites and basically consist of five carbon isoprene units, which are assembled to each other (many isoprene units) by thousands of ways. Terpenes are simple hydrocarbons, while terpenoids (monoterpenes, sesquiterpenes, diterpenes, sesterpenes, and triterpenes) are a modified class of terpenes with different functional groups and an oxidized methyl group moved or removed at various positions [56].

Organic compounds that contain the group -CHO (the aldehyde group; i.e., a carbonyl group (C=O) with a hydrogen atom bound to the carbon atom) are known as aldehydes. In systematic chemical nomenclature, aldehyde names end with the suffix -al [57].

In this study, the major components and presumably the most active components (geranial, neral, trans-2-decenal, and trans-cinnamaldehyde) of *Litsea citrata*, *Cymbopogon flexuosus*, *Coriandrum sativum*, *Cinnamomum cassia*, and *C. burmannii* EOs are aldehydes. Aldehydes are highly reactive molecules that may have a variety of effects on biological systems. Although some aldehyde-mediated effects are beneficial, many effects are deleterious, including cytotoxicity, mutagenicity, and carcinogenicity [58], and generally, they are toxic to the human body [59] and evidently, to nematodes. Despite the potential risks of aldehyde exposure, the toxic mechanisms are only understood in general terms. Human exposure to aldehydes represents a significant toxicological concern and, therefore, understanding the corresponding molecular mechanism of toxicity is important for accurate risk assessment and remediation. In this perspective, it has been shown that environmental and endogenous aldehydes can be described by their relative softness and electrophilicity, which are important electronic determinants of the respective second order reaction rates with nucleophilic targets on macromolecules. These soft-soft and hard-hard adduct reactions appear to mediate toxicity by impairing the function of macromolecules (e.g., proteins, DNA, and RNA) that play critical roles in cytophysiological processes. However, more research is needed to broaden our understanding of how these specific covalent reactions disable macromolecular targets [60].

Comparing the results for the toxicity of EOs for nematodes with a different oral apparatus, they are mostly in agreement. However, some results for *Panagrolaimus* sp. deviate from those obtained for plant parasitic nematodes.

The *Rosmarinus officinalis* (*syn. Salvia rosmarinus*) EO at 2 µg/mL induced 100% mortality of *Xiphinema index* adults [61], while in this study, the same EO was characterized as having low toxicity and classified into the first group of EOs.

The LC50 of the *M. spicata* EO was 0.2 mg/mL, for *M. javanica* juveniles [62], while in this study the LC50 of the same oil was 0.505 µL/mL against *Panagrolaimus* sp. juveniles. Good nematicidal activity against male, female and juvenile nematodes of *B. xylophilus* was achieved, among other EOs, with the essential oils of *Boswellia carterii* [31]. In this study, *Boswellia serata* was classified into the group of EOs with low toxicity. The *Pinus pinea* EO was found to be toxic against *M. incognita* juveniles with an estimated LC50 of 44 ppm [63], while in this study EOs from gymnosperms, e.g., *Pinus pinaster* (LC50: 5.078 µL/mL), generally showed low toxicity to *Panagrolaimus* species.

Variations in acute toxicity among EOs of the same plant species are greatly influenced by production, storage conditions, climatic or edaphic factors [64]. The chemical content varies even within the same crop. Significant variations were found in many EO components, both across years and throughout harvest dates within locations [65]. However, the different impact of the same EO on free-living versus plant parasitic nematodes may be due to different feeding behaviors, different dimensions, and different metabolic activities and demonstrate a possible direction in the search for active compounds that will be at the same time toxic to plant parasitic nematodes and not have unacceptable effects on the environment and non-target species.

## 4. Materials and Methods

### 4.1. Nematode Culture and Direct Contact Bioassay

A culture of *Panagrolaimus* sp. was grown monoxenically on previously frozen agricultural compost and extracted from it with a Baerman funnel [66] over 24 h. Using a compound microscope and a micropipette, juveniles were separated from adult nematodes, counted in aliquots of 50 in 20 µL of water suspension and the live specimens were used in the experiments. The 50 commercial plant EOs from 22 families were purchased from the market and used to investigate their in vitro nematicidal activity against the panagrolaimid nematode *Panagrolaimus* sp. (Table 1). Serial dilutions starting from 0.2 µL/mL, in a double decreasing range up to 0.00975 µL/mL of EOs, were made and stabilized with 0.1-µL/mL Break-Thru^®^ 446 oil enhancer. The direct contact bioassay was performed in small glass petri dishes containing 2 mL of solution and 50 nematodes incubated at 18 °C in the dark. The experiments were performed in five replicates. The lethal effect was monitored after 24 h. An aqueous solution of the emulsifier without EO served as the control. Prior to the assessment of the EOs, the mortality of panagrolaimid nematodes in the aqueous solution was compared with the mortality of nematodes in 0.1-µL/mL emulsifier and no significant differences between the treatments were observed. The nematodes were considered dead if they did not react on touching with a small needle.

### 4.2. Chemical Analyses

The gas chromatography/mass spectrometry (GC/MS) analysis was performed on an Agilent 6890N network gas chromatograph attached to a mass spectrometer (Agilent 5975B) equipped with a fused silica capillary column (HP-5ms) with dimensions as follows: 30-m length, 0.25-mm internal diameter, 0.25-μm film thickness, coated with 5% diphenyl- and 95% dimethyl-polysiloxane. The samples were diluted in diethyl ether (1:10) and a volume of 1.0 μL was injected. The injector was set at 220 °C and performed in the split mode at a ratio of 1:20. Helium was used as the carrier gas at a flow rate of 0.9 mL/min. The oven temperature increased from 60 to 246 °C at a rate of 3 °C/min. Temperatures of the mass selective detector (MSD) transfer line, ion source and quadruple mass analyzer were set at 280, 230 and 150 °C, respectively. The ionization voltage was 70 eV and the scan range was 35–400 *m*/*z*.

Compound identifications were based on comparisons of their mass spectra with the mass spectra obtained from the National Institute of Standards and Technology database and by comparisons of the retention indices with values reported in the literature (RI^lit^) [67]. A homologous series of *n*-alkanes (C_8_–C_34_) was run under the same operating conditions as the EO to determine the experimental retention indices (RI^exp^). The relative amounts of individual components (expressed in percentages) were calculated via peak area normalization, without the use of correction factors. Compounds present in traces (tr) with their amounts less than 0.05% are indicated (Table 2, Table 3, Table 4, Table 5, Table 6 and Table 7).

### 4.3. Statistical Data Analysis

In order to evaluate the nematicidal activity of the EOs, median lethal concentration (LC50) was calculated using the Probit Analysis program [68]. The *Panagrolaimus* mortality was corrected using Abbott’s formula [69]. The nematicidal activity, i.e., acute toxicity of the examined EOs based on the median lethal concentration, was designated as high (LC50: <0.1 µL/mL), moderate (LC50: 0.1–1 µL/mL) and low (LC50: >1 µL/mL) (Table 1).

## Figures and Tables

**Table 1:** LC50 (in µL/mL with 95% confidence limits and the slope values) of 50 plant essential oils investigated on *Panagrolaimus* sp. juveniles.

Species Name	Plant Part	Family	LC50 (95% CL)	Slope
*Pogostemon cablin*	leaves	Lamiaceae	5.641 (3.18–17.10)	2.14
*Pinus pinaster*	needles	Pinaceae	5.078 (3.38–9.65)	1.65
*Santalum album*	wood	Santalaceae	4.781 (3.45–6.34)	2.12
*Azadirachta indica*	seeds	Meliaceae	4.482 (2.52–10.01)	2.02
*Boswellia serrata*	resin	Burseraceae	4.394 (3.44–5.53)	3.03
*Commiphora myrrha*	resin	Burseraceae	4.301 (2.81–6.98)	2.79
*Juniperus virginiana*	wood	Juniperaceae	3.782 (2.43–5.48)	1.81
*Cupressus sempervirens*	needles	Cupressaceae	3.360 (2.47–4.82)	1.85
*Abies sibirica*	needles	Pinaceae	3.269 (2.14–5.41)	1.58
*Cedrus atlantica*	wood	Pinaceae	2.943 (2.04–4.43)	1.62
*Juniperus communis*	berries	Juniperaceae	2.513 (1.64–3.83)	1.68
*Eucalyptus globulus*	leaves	Myrtaceae	1.994 (1.47–2.56)	3.70
*Myrtus communis*	leaves	Myrtaceae	1.933 (1.34–2.65)	2.27
*Piper nigrum*	peppercorns	Piperaceae	1.775 (1.37–2.25)	2.37
*Petroselinum crispum*	seeds	Apiaceae	1.704 (1.14–2.38)	4.08
*Zingiber officinale*	roots	Zingiberaceae	1.633 (1.28–2.09)	1.98
*Turnera diffusa*	leaves/flowers	Passifloraceae	1.550 (1.14–2.04)	3.11
*Abies alba*	needles	Pinaceae	1.444 (1.03–1.95)	1.87
*Taxandria fragrans*	leaves	Myrtaceae	1.437 (0.99–1.93)	2.49
*Melaleuca alternifolia*	leaves	Myrtaceae	1.150 (0.76–1.57)	4.83
*Vanilla planifolia*	beans	Orchidaceae	1.135 (0.83–1.50)	2.08
*Salvia rosmarinus*	leaves/flowers	Lamiaceae	1.128 (0.88–1.41)	3.04
*Curcuma longa*	rhizomes	Zingiberaceae	1.116 (0.70–1.62)	1.50
*Lavandula* sp.	leaves/flowers	Lamiaceae	0.810 (0.62–1.01)	3.49
*Laurus nobilis*	leaves	Lauraceae	0.594 (0.31–0.92)	4.80
*Melaleuca quinquenervia*	leaves	Myrtaceae	0.593 (0.40–0.82)	1.76
*Origanum vulgare*	leaves/flowers	Lamiaceae	0.508 (0.38–0.65)	3.33
*Mentha spicata*	leaves/flowers	Lamiaceae	0.505 (0.39–0.64)	2.78
*Pimpinella anisum*	seeds	Apiaceae	0.450 (0.26–0.63)	4.87
*Salvia sclarea*	leaves/flowers	Lamiaceae	0.430 (0.31–0.57)	1.81
*Anethum graveolens*	seeds	Apiaceae	0.428 (0.32–0.56)	3.00
*Mentha piperita*	leaves/flowers	Lamiaceae	0.405 (0.23–0.58)	3.93
*Thymus vulgaris*	leaves/flowers	Lamiaceae	0.391 (0.29–0.50)	2.22
*Gaultheria procumbens*	leaves	Ericaceae	0.367 (0.26–0.48)	2.25
*Myristica fragrans*	seeds	Myristicaceae	0.345 (0.23–0.48)	4.24
*Pelargonium asperum*	leaves	Geraniaceae	0.279 (0.20–0.37)	3.03
*Cymbopogon martini*	grass blades	Poaceae	0.275 (0.20–0.35)	4.06
*Syzygium aromaticum*	buds	Myrtaceae	0.272 (0.20–0.34)	3.91
*Ocimum basilicum*	leaves/flowers	Lamiaceae	0.263 (0.19–0.35)	2.65
*Uncaria tomentosa*	bark	Rubiaceae	0.222 (0.17–0.29)	2.47
*Illicium verum*	seeds	Schisandraceae	0.191 (0.14–0.25)	2.94
*Cinnamomum verum*	leaves	Lauraceae	0.172 (0.12–0.23)	5.00
*Cananga odorata*	flowers	Annonaceae	0.145 (0.10–0.19)	5.00
*Mellisa officinalis*	leaves	Lamiaceae	0.124 (0.09–0.16)	2.74
*Litsea citrata*	fruits	Lauraceae	0.091 (0.06–0.12)	3.14
*Foeniculum vulgare*	seeds	Apiaceae	0.080 (0.05–0.11)	3.68
*Cymbopogon flexuosus*	grass blades	Poaceae	0.071 (0.05–0.09)	2.92
*Coriandrum sativum*	leaves	Apiaceae	0.044 (0.02–0.04)	3.95
*Cinnamomum cassia*	bark	Lauraceae	0.034 (0.02–0.04)	2.00
*Cinnamomum burmanii*	bark	Lauraceae	0.033 (0.02–0.04)	2.73

**Table 2:** Chemical composition of *Litsea citrata* essential oil (EO).

RT *	Compound	Molecular Formula	RI^exp^ **	RI^lit^ ***	%
18.23	Geranial (=E–citral)	C_10_H_16_O	1274	1267	43.4
16.94	Neral (=Z-citral)	C_10_H_16_O	1243	1238	32.2
8.49	Limonene	C_10_H_16_	1028	1029	9.4
8.57	1,8-Cineole	C_10_H_18_O	1030	1031	1.9
24.27	(E)-Caryophyllene	C_15_H_24_	1419	1419	1.6
13.17	Citronellal	C_10_H_18_O	1153	1153	1.4
11.03	Linalool	C_10_H_18_O	1100	1096	1.2
5.70	α-Pinene	C_10_H_16_	933	939	1.1
14.40	(E)-Isocitral	C_10_H_16_O	1183	1180	1.1
17.42	Geraniol	C_10_H_18_O	1255	1252	0.9
6.87	β-Pinene	C_10_H_16_	977	979	0.8
7.10	6-Methyl-5-hepten-2-one	C_8_H_14_O	985	985	0.8
13.63	(Z)-Isocitral	C_10_H_16_O	1164	1164	0.8
6.76	Sabinene	C_10_H_16_	973	975	0.6
14.71	α-Terpineol	C_10_H_18_O	1190	1188	0.6
16.31	Nerol	C_10_H_18_O	1229	1229	0.4
6.10	Camphene	C_10_H_16_	948	954	0.3
7.25	Dehydro-1,8-cineole	C_10_H_16_O	991	991	0.2
23.10	β-Elemene	C_15_H_24_	1392	1390	0.2
12.84	exo-Isocitral	C_10_H_16_O	1144	1144	0.1
14.17	Terpinen-4-ol	C_10_H_18_O	1177	1177	0.1
25.65	α-Humulene	C_15_H_24_	1453	1454	0.1
5.51	α-Thujene	C_10_H_16_	926	930	tr
8.35	o-Cymene	C_10_H_14_	1024	1026	tr
Total identified					99.2

* RT—retention time, ** RI^exp^—retention index obtained experimentally, *** RI^lit^—retention index from the literature, tr—traces.

**Table 3:** Chemical composition of *Foeniculum vulgare* EO.

RT *	Compound	Molecular Formula	RI^exp^ **	RI^lit^ ***	%
18.93	(E)-Anethole	C_10_H_12_O_2_	1291	1284	74.3
11.28	α-Pinene oxide	C_10_H_16_O	1106	1099	9.0
15.06	Methyl chavicol (=Estragol)	C_10_H_12_O	1199	1196	2.9
5.71	α-Pinene	C_10_H_16_	933	939	2.3
8.48	Limonene	C_10_H_16_	1028	1029	2.3
10.62	Fenchone	C_10_H_16_O	1088	1086	2.1
16.94	Carvone	C_10_H_14_O	1244	1243	2.1
17.39	p-Anis aldehyde	C_8_H_8_O_2_	1254	1250	1.3
8.33	o-Cymene	C_10_H_14_	1024	1026	0.7
12.81	Camphor	C_10_H_16_O	1144	1146	0.6
8.57	1,8-Cineole	C_10_H_18_O	1030	1031	0.5
11.07	Linalool	C_10_H_18_O	1001	1096	0.4
9.53	γ-Terpinene	C_10_H_16_	1058	1059	0.3
6.10	Camphene	C_10_H_16_	948	954	0.1
6.87	β-Pinene	C_10_H_16_	977	979	0.1
11.79	α-Campholenal	C_10_H_16_O	1119	1126	0.1
6.76	Sabinene	C_10_H_16_	973	975	tr
7.24	Myrcene	C_10_H_16_	991	990	tr
Total identified					99.1

* RT—retention time, ** RI^exp^—retention index obtained experimentally, *** RI^lit^—retention index from the literature, tr—traces.

**Table 4:** Chemical composition of *Cymbopogon flexuosus* EO.

RT *	Compound	Molecular Formula	RI^exp^ **	RI^lit^ ***	%
18.24	Geranial (=E-citral)	C_10_H_16_O	1274	1267	40.3
16.94	Neral (=Z-citral)	C_10_H_16_O	1244	1238	30.9
22.86	Geranyl acetate	C_12_H_20_O_2_	1385	1381	5.4
17.45	Geraniol	C_10_H_18_O	1255	1252	4.5
7.10	6-Methyl-5-hepten-2-one	C_8_H_14_O	985	985	2.7
24.27	(E)-Caryophyllene	C_15_H_24_	1419	1419	2.1
11.03	Linalool	C_10_H_18_O	1101	1096	1.4
28.08	γ-Cadinene	C_15_H_24_	1514	1513	1.4
14.38	(E)-Isocitral	C_10_H_16_O	1182	1180	1.3
6.10	Camphene	C_10_H_16_	948	954	1.1
8.48	Limonene	C_10_H_16_	1028	1029	1.1
10.00	4-Nonanone	C_9_H_18_O	1071	-	0.9
13.64	(Z)-Isocitral	C_10_H_16_O	1164	1164	0.9
5.71	α-Pinene	C_10_H_16_	933	939	0.3
7.24	1,8-Dehydro-cineole	C_10_H_16_O	991	990	0.3
8.56	1,8-Cineole	C_10_H_18_O	1030	1031	0.3
13.03	trans-α-Necrodol	C_10_H_18_O	1149	1148	0.3
13.16	Citronellal	C_10_H_18_O	1152	1153	0.3
28.45	δ-Cadinene	C_15_H_24_	1523	1523	0.3
30.72	Caryophyllene oxide	C_15_H_24_O	1582	1582	0.3
8.76	(Z)-β-Ocimene	C_10_H_16_	1036	1037	0.2
25.64	α-Humulene	C_15_H_24_	1453	1454	0.2
5.43	Tricyclene	C_10_H_16_	922	926	0.1
8.34	o-Cymene	C_10_H_14_	1024	1026	0.1
9.14	(E)-β-Ocimene	C_10_H_16_	1046	1050	0.1
10.62	Terpinolene	C_10_H_16_	1088	1088	0.1
12.84	exo-Isocitral	C_10_H_16_O	1144	1144	0.1
14.08	Rosefuran epoxide	C_10_H_14_O_2_	1175	1177	0.1
14.72	α-Terpineol	C_10_H_18_O	1191	1188	0.1
16.32	Nerol	C_10_H_18_O	1229	1229	0.1
6.87	β-Pinene	C_10_H_16_	977	979	tr
7.60	n-Octanal	C_8_H_16_O	1003	998	tr
Total identified					97.3

* RT—retention time, ** RI^exp^—retention index obtained experimentally, *** RI^lit^—retention index from the literature, tr—traces.

**Table 5:** Chemical composition of *Coriandrum sativum* EO.

RT *	Compound	Molecular Formula	RI^exp^ **	RI^lit^ ***	%
17.78	(2E)-Decenal	C_10_H_18_O	1263	1263	28.2
18.10	(2E)-Decen-1-ol	C_10_H_20_O	1271	1271	16.3
11.08	Linalool	C_10_H_18_O	1101	1096	18.4
26.20	(2E)-Dodecenal	C_12_H_22_O	1467	1466	8.8
15.35	n-Decanal	C_10_H_20_O	1206	1201	6.2
18.19	n-Decanol	C_10_H_22_O	1273	1269	5.4
34.07	n-Tetradecanol	C_14_H_30_O	1673	1672	4.0
21.97	(2E)-Undecenal	C_11_H_20_O	1363	1360	1.7
12.80	Camphor	C_10_H_16_O	1143	1146	1.2
23.85	Dodecanal	C_12_H_24_O	1409	1408	1.1
8.34	o-Cymene	C_10_H_14_	1024	1026	0.9
5.71	α-Pinene	C_10_H_16_	933	939	0.7
15.00	(4E)-Decenal	C_10_H_18_O	1198	1196	0.6
17.43	Geraniol	C_10_H_18_O	1255	1252	0.6
10.03	cis-Linalool oxide	C_10_H_18_O_2_	1072	1072	0.4
10.62	trans-Linalool oxide	C_10_H_18_O_2_	1088	1086	0.4
19.62	Undecanal	C_11_H_22_O	1307	1306	0.4
22.85	Geranyl acetate	C_12_H_20_O_2_	1384	1381	0.4
7.60	n-Octanal	C_8_H_16_O	1003	998	0.3
8.48	Limonene	C_10_H_16_	1028	1029	0.3
31.86	Tetradecanal	C_14_H_28_O	1613	1612	0.3
8.57	1,8-Cineole	C_10_H_18_O	1030	1031	0.2
9.53	γ-Terpinene	C_10_H_16_	1058	1059	0.2
14.85	(4Z)-Decenal	C_10_H_18_O	1194	1194	0.2
6.10	Camphene	C_10_H_16_	948	954	0.1
6.88	β-Pinene	C_10_H_16_	977	979	0.1
14.71	α-Terpineol	C_10_H_18_O	1190	1188	0.1
13.69	Borneol	C_10_H_18_O	1165	1169	tr
14.17	Terpinen-4-ol	C_10_H_18_O	1177	1177	tr
Total identified					97.5

* RT—retention time, ** RI^exp^—retention index obtained experimentally, *** RI^lit^—retention index from the literature, tr—traces.

**Table 6:** Chemical composition of *Cynnamomum cassia* EO.

RT *	Compound	Molecular Formula	RI^exp^ **	RI^lit^ ***	%
24.54	(E)-Cinnamaldehyde	C_9_H_8_O	1272	1267	76.7
33.02	Eugenol acetate	C_12_H_14_O_3_	1535	1521	7.4
32.39	δ-Cadinene	C_15_H_24_	1517	1522	6.2
29.95	(E)-Cinnamyl acetate	C_11_H_12_O_2_	1448	1443	4.0
17.88	Phenethyl alcohol	C_8_H_10_O	1126	1106	0.8
11.96	Benzaldehyde	C_7_H_6_O	962	952	0.7
21.91	(Z)-Cinnamaldehyde	C_9_H_8_O	1220	1217	0.6
22.78	Carvone	C_10_H_14_O	1243	1239	0.4
19.69	Hydrocinnamaldehyde	C_9_H_10_O	1163	1163	0.3
25.28	α-Methylcinnamaldehyde	C_10_H_10_O	1332	1318	0.3
30.51	Coumarin	C_9_H_6_O_2_	1456	1432	0.3
15.10	γ-Terpinene	C_10_H_16_	1056	1054	0.2
23.20	2-Phenyl ethyl acetate	C_10_H_12_O_2_	1259	1254	0.2
27.57	α-Copaene	C_15_H_24_	1368	1374	0.2
10.93	α-Pinene	C_10_H_16_	929	932	0.1
10.93	Camphene	C_10_H_16_	943	946	0.1
14.39	1,8-Cineole	C_10_H_18_O	1027	1026	0.1
19.88	Borneol	C_10_H_18_O	1168	1165	0.1
20.19	Terpinen-4-ol	C_10_H_18_O	1174	1174	0.1
26.98	Eugenol	C_10_H_12_O_2_	1359	1356	0.1
30.99	γ-Muurolene	C_15_H_24_	1470	1478	0.1
31.11	β-Selinene	C_15_H_24_	1478	1489	0.1
32.51	trans-Cadina-1,4-diene	C_15_H_24_	1526	1533	0.1
25.90	1-epi-Cubenol	C_15_H_26_O	1622	1627	0.1
12.48	β-Pinene	C_10_H_16_	973	974	tr
14.25	β-Phellandrene	C_10_H_16_	1025	1025	tr
16.93	Terpinolene	C_10_H_16_	1087	1086	tr
27.27	Cyclosativene	C_15_H_24_	1357	1358	tr
28.10	Sativene	C_15_H_24_	1382	1374	tr
28.84	Isosativene	C_15_H_24_	1401	1417	tr
29.14	(E)-Caryophyllene	C_15_H_24_	1413	1417	tr
31.76	α-Muurolene	C_15_H_24_	1494	1500	tr
Total identified					99.3

* RT—retention time, ** RI^exp^—retention index obtained experimentally, *** RI^lit^—retention index from the literature, tr—traces.

**Table 7:** Chemical composition of *Cynnamomum burmannii* EO.

RT *	Compound	Molecular Formula	RI^exp^ **	RI^lit^ ***	%
24.51	(E)-Cinnamaldehyde	C_9_H_8_O	1272	1267	80.5
20.70	α-Terpineol	C_10_H_18_O	1189	1186	1.9
32.51	δ-Cadinene	C_15_H_24_	1517	1522	1.7
27.57	α-Copaene	C_15_H_24_	1368	1374	1.5
21.82	(Z)-Cinnamaldehyde	C_9_H_8_O	1220	1217	1.5
19.60	Hydrocinnamaldehyde	C_9_H_10_O	1163	1163	1.3
19.79	Borneol	C_10_H_18_O	1168	1165	1.2
20.17	Terpinen-4-ol	C_10_H_18_O	1174	1174	1.2
31.74	α-Muurolene	C_15_H_24_	1494	1500	1.2
29.05	(E)-Caryophyllene	C_15_H_24_	1413	1417	0.8
17.10	Linalool	C_10_H_18_O	1100	1095	0.5
11.87	Benzaldehyde	C_7_H_6_O	962	952	0.4
24.54	Safrole	C_10_H_10_O_2_	1288	1285	0.4
28.76	Isosativene	C_15_H_24_	1401	1417	0.4
31.32	α-Selinene	C_15_H_24_	1487	1498	0.4
24.75	Tridecane	C_13_H_28_	1295	1300	0.3
14.29	β-Phellandrene	C_10_H_16_	1025	1025	0.2
15.84	cis-Linalool oxide	C_10_H_18_O_2_	1071	1067	0.2
20.54	Cryptone	C_9_H_14_O	1185	1183	0.2
28.03	Sativene	C_15_H_24_	1382	1374	0.2
30.92	γ-Muurolene	C_15_H_24_	1470	1478	0.2
31.15	β-Selinene	C_15_H_24_	1478	1489	0.2
30.21	(E)-Cinnamyl acetate	C_11_H_12_O_2_	1448	1443	0.2
36.26	epi-α-Murrolol	C_15_H_26_O	1639	1640	0.2
13.37	α-Phellandrene	C_10_H_16_	1002	1002	0.1
14.42	1,8-Cineole	C_10_H_18_O	1027	1026	0.1
14.96	γ-Terpinene	C_10_H_16_	1056	1054	0.1
16.62	p-Cymenene	C_10_H_14_	1087	1089	0.1
16.99	Terpinolene	C_10_H_16_	1087	1086	0.1
18.87	trans-Limonene oxide	C_10_H_16_O	1137	1137	0.1
25.24	α-Methylcinnamaldehyde	C_10_H_10_O	1332	1318	0.1
27.18	Cyclosativene	C_15_H_24_	1357	1358	0.1
28.25	β-Elemene	C_15_H_24_	1386	1389	0.1
28.54	(Z)-Caryophyllene	C_15_H_24_	1400	1408	0.1
29.82	Humulene	C_15_H_24_	1446	1452	0.1
30.51	Coumarin	C_9_H_6_O_2_	1456	1432	0.1
36.35	α-Muurolol (Torreyol)	C_15_H_26_O	1643	1644	0.1
12.83	Myrcene	C_10_H_16_	994	998	tr
17.70	Phenethyl alcohol	C_8_H_10_O	1126	1106	tr
19.45	Isoborneol	C_10_H_18_O	1154	1155	tr
32.80	trans-Cadina-1,4-diene	C_15_H_24_	1526	1533	tr
33.15	α-Calacorene	C_15_H_20_	1538	1544	tr
32.92	Benzyl benzoate	C_14_H_12_O_2_	1772	1759	tr
Total identified					98.1

* RT—retention time, ** RI^exp^—retention index obtained experimentally, *** RI^lit^—retention index from the literature, tr—traces.
